# Relationship between Sleep Quality and Shoulder Disorders in People with Stroke

**DOI:** 10.3390/medicina59112010

**Published:** 2023-11-15

**Authors:** Manal M. Al Battat, Mohammad Etoom, Alia A. Alghwiri

**Affiliations:** 1 Humanity and Inclusion Organization, Amman 11195, Jordan; manal_albattat@yahoo.com; 2 Department of Rehabilitation Sciences, Faculty of Applied Medical Sciences, Jordan University of Science and Technology, Irbid 22110, Jordan; msetoom@just.edu.jo; 3 Department of Physiotherapy, School of Rehabilitation Sciences, The University of Jordan, Amman 11942, Jordan

**Keywords:** sleeping, upper limb, cerebrovascular accident, cross-sectional study, non-motor outcome

## Abstract

*Background and objective*: The primary aim of the study was to investigate the relationship between sleep quality (SQ) and shoulder disorders in people after a stroke. The secondary aim was to explore the relationship between SQ and stroke outcomes, including the impact of stroke, fatigue, and quality of life (QOL). *Material and Methods*: A sample of 94 stroke patients was included. The Pittsburgh Sleep Quality Index (PSQI) was used to assess SQ, whereas the Shoulder Pain and Disability Index (SPADI) was utilized to assess shoulder pain and disability. The Stroke Impact Scale 16, the Modified Fatigue Impact Scale, and the Medical Outcomes Study Short Form 12 were also used as secondary measures. *Results*: The prevalence of poor SQ, shoulder pain, and shoulder disability in our sample was 60%, 78%, and 85%, respectively. The global PSQI score had a significant association with the SPADI pain subscale. There were some significant correlations between the PSQI subscales and shoulder pain and disability. The PSQI significantly correlated with stroke recovery, fatigue, and QOL. The daytime dysfunction PSQI subscale was significantly correlated with all shoulder and stroke outcomes and their subscale. *Conclusion*: SQ was associated more with the non-motor outcomes of stroke, including pain, cognitive fatigue, and mental status, than the motor outcomes. Considering SQ during upper extremity rehabilitation and care processes is essential.

## 1. Introduction

Stroke is a common neurological disorder that has several motor and non-motor complications [1]. Upper extremity impairments, muscle tone abnormalities, and balance and gait disturbances are common motor disorders associated with stroke [2]. Common non-motor disorders include pain, cognitive dysfunctions, psychological problems, fatigue, and sleep disorders [3].

Upper extremity impairments are among the most common motor defects after stroke [4,5]. About 30–66% of stroke survivors reported persistent movement impairment in their affected arm in daily life and 15–30% of stroke survivors experience long-lasting hemiparesis in the affected arm [6]. Shoulder pain and disability are UE impairments, which affect the performance of activities of daily living (ADL) and the quality of life (QoL) of patients after stroke [7]. Shoulder subluxation/dislocation, shoulder impingement, adhesive capsulitis, and decreased shoulder range of motion (ROM) are associated factors of shoulder pain and disability post-stroke that may impair upper extremity post-stroke recovery [8]. In addition to the previous motor factors, non-motor factors, such as cognitive abilities, have been found that may impair upper extremity post-stroke recovery [9]. There is a need to investigate the relationship between shoulder impairments and the non-motor features of stroke. 

Sleep is a health need for humans. Sleep quality (SQ) has been found to be impaired in people after they have had a stroke [10]. Pasic et al., (2011) studied the prevalence of sleep disorders among patients with stroke and they found that 78% of them had sleep disorders with various degrees of severity [11]. Fragmentation of sleep, insomnia, hypersomnia, breathing disorders, and sleep-related movement disorders were reported as common sleep complications after stroke [12]. Furthermore, recent analyses found long-term impairments in rapid eye movement sleep and the sleep–wake cycle in stroke patients [13,14]. Poor SQ was found to have serious consequences that affect normal ADL and QOL in people with stroke [11]. Poor SQ may impair stroke recovery, including upper extremity recovery [15].

One of the factors that can worsen SQ is upper extremity impairments [16]. Studies show that the hyperarousal state of shoulder pain can negatively affect SQ [16]. Furthermore, the inflammatory mediators from shoulder muscles, especially rotator calf muscles, contribute to poor SQ [17]. In stroke, the current evidence shows a relationship between shoulder impairments and sleep [18]. It was found that sleep parameters were significantly worse in patients who have shoulder pain [19], or shoulder musculoskeletal impairments, such as biceps tenosynovitis [20], after stroke. However, the studies did not investigate the relationship between SQ and shoulder disability or function. Therefore, there is a need for more studies that clarify the relationship between SQ and shoulder disorders in people with stroke. In the current study, we aimed to investigate the relationship between SQ and shoulder pain/disability in people after a stroke, and to explore the relationship of SQ with other stroke outcomes, including the impact of stroke, fatigue, and QOL.

## 2. Materials and Methods

### 2.1. Study Design

The study design was a cross-sectional quantitative study. The study was reported according to the Strengthening the Reporting of Observational Studies in Epidemiology (STROBE) guidelines [21]. The ethical approval for the study was granted by the scientific research committee at the University of Jordan (15/2020/864) in 11 March 2020.

### 2.2. Setting, Participants, and Sampling

Patients were invited to participate in this study, using a registry list from the Humanity and Inclusion (HI) organization in Jordan, between December 2020 and March 2021. The eligibility criteria included any participant who was 18 years and older and had a confirmed diagnosis of stroke, irrespective of gender, or stroke type. The participants who were diagnosed with any sleep disorder or shoulder disorder prior to stroke, neurological disorders other than stroke, or the use of sleep medications or other interventions interfering with sleep and sleepiness, were excluded from the study. 

### 2.3. Assessments and Outcome Measures

#### 2.3.1. Demographics, Medical History, and Stroke Characteristics including Age, Gender, Refugee Status, Body Mass Index, Education Level, Marital Status, Smoking, Presence of Hypertension, Diabetes Mellitus, Heart Disease or Cholesterol

The stroke characteristics were a first or recurrent stroke, the time since the last stroke, and the stroke side. All the information was extracted from the registry. All the study outcomes were filled out online by the participants. 

#### 2.3.2. Pittsburgh Sleep Quality Index (PSQI)

The PSQI is a self-reported questionnaire that is used to assess SQ during a previous month. The questionnaire consists of 19 items that are included under 7 components [22]. These components are subjective SQ, sleep latency, sleep duration, sleep efficiency, sleep disturbance, use of sleeping medication, and daytime dysfunction. The cut-off score for the PSQI is 6 and above for poor sleepers. It was found to be a valid and reliable tool to assess SQ [23], and is frequently used in stroke research [12]. This study used the Arabic version of the PSQI, which has been translated and validated [24]. 

#### 2.3.3. Shoulder Pain and Function Measured by Shoulder Pain Disability Index (SPADI)

The SPADI is a 13-item self-reported questionnaire that is used to assess shoulder disorders, including disability (8 items) and pain (5 items) [25]. A standardized formula is used to form the final score of 100 for each SPADI subscale. The score ranges from 0% to 100%, with a high score indicating worse disability or pain. The cut-off score for the SPADI is 50% and above, from the standardized score of each subscale [26]. The SPADI has good psychometric properties [26], and has been used in stroke research [20,27]. We used an Arabic version of the SPADI, which had been previously translated and validated [28].

#### 2.3.4. Stroke Outcomes

(i). The Stroke Impact Scale version 3 (SIS V. 3.0) is a questionnaire that is used to evaluate the impact of stroke on overall health-related well-being [29]. The Arabic version was used to include 6 subscales that covered ADL, memory, emotions, communication, social participation, and stroke recovery. The SIS has good psychometric properties [29].
(ii). The Modified Fatigue Impact Scale (MFIS) is a 21-item self-reported questionnaire that measures the impact of different types of fatigue, including physical, cognitive, and psychosocial abilities [30]. The MFIS is used to assess fatigue in stroke [31], and is translated into the Arabic language [32].
(iii). The Medical Outcomes Study Short Form 12 (SF-12) is a 12-item questionnaire that assesses QOL through physical and mental components. The SF-12 has good psychometric properties in stroke populations [33]. The SF-12 was translated and validated in the Arabic language, which was used in this study [34].

### 2.4. Sample Size Calculation

A priori power analysis was conducted using the software package, GPower, with a power of 0.8 and alpha of 0.05, and an effect size of 0.33 for the PSQI [35], indicating that a sample size of 67 participants would be sufficient to evaluate the correlation [36].

### 2.5. Statistical Analysis

Descriptive statistics, including the mean, standard deviation (SD), frequency, and percentages, were calculated. The differences between a good and poor sleeper through the patient’s demographics, medical history, and stroke characteristics were analyzed through the Kruskal–Wallis test, or the Chi-square test, with a post hoc Mann–Whitney test, or Fisher’s exact tests, respectively. The use of the abovementioned non-parametric tests was due to the non-normal distribution of the data. Spearman’s rank (rho) correlation analysis was used to assess the association between the PSQI and its components (dependent variables) and the SPADI, SIS, MFIS, and SF-12 (independent variables). All study analyses were conducted at a 95% confidence interval and with *p* ≤ 0.05, using the Statistical Package for the Social Sciences (SPSS) version 23. IBM Corp (Armonk, NY, USA).

## 3. Results

### 3.1. Sample Characteristics

Of the 127 participants initially screened, 94 participants met the inclusion criteria and were included. Fifty-six participants (60%) were classified as poor sleepers. The ages ranged between 27 and 85 years, and 44.7% of the participants were females. The time since stroke ranged between 1 to 360 months, while 22 participants had a recurrent stroke. Table 1 shows the demographics, past medical history, and stroke characteristics of the study sample, which did not significantly differ between good and poor sleepers.

### 3.2. PSQI

The mean (SD) of the PSQI global score was 6.87 (3.05) and around 60% of the study sample had poor SQ. The most impaired PSQI domain was sleep disturbance with a mean (SD) of 1.65 (0.60), followed by daytime dysfunction 1.54 (0.8), sleep latency 1.26 (1.11), subjective SQ 1.17 (0.85), sleep duration 0.78 (1.15), and the use of sleep promoting medications 0.28 (0.74), while the habitual sleep efficiency component was the least impaired PSQI item 0.2 (0.63).

### 3.3. Shoulder Disorders

The mean (SD) total score of the SPADI was 73.25 (18.98). The mean (SD) of the SPADI disability subscale was 82.67 (25.89), which was higher than the SPADI pain subscale at 63.83 (21.24). The frequency of shoulder pain and disability was 78% and 85%, respectively, in the study sample.

### 3.4. The Correlation between SQ and Shoulder Disorders

Table 2 shows the correlation between the global and domain scores of the PSQI and subscale scores of the SPADI. There was no significant correlation between the global score of the PSQI and the SPADI total score (r = 0.06, *p* = 0.57). The PSQI global score was significantly correlated with the SPADI pain subscale (r = 0.27, *p* < 0.01), but not with the SPADI disability subscale (r = 0.06, *p* = 0.57). 

The pain subscale of the SPADI was also significantly correlated with the following domains in the PSQI: subjective SQ (r = 0.35, *p* < 0.01), sleep disturbance (r = 0.38, *p* < 0.01), and daytime dysfunction (r = 0.37, *p* = 0.001). The disability subscale of the SPADI had significant correlations with sleep duration (r = 0.21, *p* = 0.04) and daytime dysfunction (r = 0.25, *p* = 0.02). For the total score of the SPADI, it was significantly correlated with the subjective SQ (r = 0.28, *p* = 0.01), sleep disturbance (r = 0.36, *p* = 0.01), and daytime dysfunction (r = 0.30, *p* = 0.01) domains in the PSQI. Daytime dysfunction was significantly correlated with both the SPADI pain and SPADI disability subscales.

### 3.5. The Correlation between QOS and Stroke Outcomes (Impact of Stroke, Fatigue, and QOL)

The PSQI global score was significantly correlated with the stroke recovery subscale of the SIS, and with physical, cognitive, psychosocial, and total scores of the MFIS. Mental SF-12 and the total score of SF-12 were significantly correlated with the PSQI global score, but not the physical SF-12 (Table 3). Table 3 also explains the correlation between the PSQI components and stroke outcomes. Daytime dysfunction was correlated with all the included stroke outcomes and their subscales.

## 4. Discussion

This study aimed to investigate the relationship between sleep quality and shoulder disorders, including pain and disability, among stroke patients, through a cross-sectional observation study. The study found some relationships between sleep and shoulder disorders, mainly pain, among stroke patients. Poor SQ was highly prevalent; 60% of the participants were categorized with poor sleep quality. The high prevalence of poor SQ in our study can be explained by two reasons. First, the study was conducted during the COVID-19 pandemic. Poor SQ was worse in different populations during the COVID-19 pandemic [37]. Second, 57.4% of the study participants were refugees, and 67% of them were categorized as poor sleepers. Previous studies show that sleep disorders and poor SQ are more common among refugees [38]. Previous articles showed a varied prevalence of poor sleepers, ranging between 23% to 64%, among different stroke populations [12,39]. In this study, SQ did not significantly differ according to the participants characteristics, the time since the last stroke, or the stroke side. Previous studies found that a subcortical stroke and cognitive impairments were determinants for poor SQ [40,41]. 

Sleep was related to shoulder disorders, including pain and disability. Shoulder disorders were assessed via the SPADI using the shoulder for different activities and the related pain. Previous studies found that poor SQ is associated with impaired motor function [42]. A biomechanical analysis found that SQ can affect motor performance [43]. In neurologic populations, poor SQ negatively affects motor skills acquisition [44] and motor learning [45], which impairs motor recovery. In the current study, sleep was correlated significantly with stroke recovery. Therefore, we suggest that poor SQ can impair upper extremity recovery and, therefore, stroke recovery. On the other hand, the non-use of the hemiplegic upper extremity after stroke due to shoulder disorders and plegia may worsen SQ. Decreased limb movement and ascending neuronal activity can contribute to sleep disorders by decreasing the activity of the frontal cortices. Hypofunction in the frontal cortical areas was found in people with sleep disorders [46]. Therefore, we think that the relationship between sleep disorders and shoulder disability is bidirectional. Sleep impairments reduce pain tolerance [47]. Dopamine and opioid system deficiencies can also reduce pain tolerance in stroke [48]. 

SQ was correlated significantly with fatigue and SF-36. Specifically, SQ was related to cognitive fatigue more than physical fatigue, and with mental more than physical SF-12, as well as with shoulder pain more than shoulder disability. These relationships suggest that SQ was more related to non-motor factors of shoulder disorders and stroke recovery than motor factors. Daytime dysfunction was correlated with all the included stroke outcomes and their subscales. Furthermore, it was significantly correlated with shoulder pain and disability. Excessive daytime sleepiness is a prevalent symptom among stroke survivors. It is an independent risk factor for stroke and increases the risk of recurrent stroke [11]. 

The study findings on the relationship between SQ and shoulder disorder imply clinical and research recommendations. Consideration of SQ during upper extremity rehabilitation and care processes is important. SQ can affect shoulder rehabilitation outcomes [49]. Clinical trials have found that a number of interventions can improve both sleep and upper extremity impairments. The interventions include aerobic exercise [50] and cognitive behavioral therapy [51]. Future research should investigate the effect of the current upper extremity interventions on SQ. The effectiveness of motor rehabilitation interventions on sleep should be investigated in order to find the best upper extremity interventions. 

Our study had several limitations. All the measures were subjective or self-reported measures. The use of objective measures would have added a clearer idea about the level of functioning and disability in our sample [52]. The study assessed the severity of shoulder pain, mainly musculoskeletal pain. Previous research [53] has found that pain in people with Parkinson’s disease and poor SQ was mainly radiating, akathisic, and paresthetic in type. Therefore, more research is needed to assess the types of shoulder pain in stroke that may be more related to poor SQ. 

## 5. Conclusions

Some correlations between SQ and shoulder disorders were evident, especially between SQ and shoulder pain in people with stroke. SQ was associated more with non-motor outcomes, including pain, cognitive fatigue, and mental QOL, than motor outcomes of stroke. SQ should be considered during the assessment and management of the upper extremities in people with stroke. Furthermore, tailored and comprehensive intervention programs that consider both motor and non-motor factors would be more successful in the rehabilitation of people after stroke.

## Figures and Tables

**Table 1:** Demographics, medical history, and stroke characteristics of the study sample.

Variable	All Participants (*n* = 94)	Good Sleeper (*n* = 38)	Poor Sleeper (*n* = 56)	*p* Value
Age, mean ± SD	57.27 ± 11.41	57.5 ± 11.89	57.1 ± 11.17	0.778
Gender, *n* (%)				
Male	52 (55.3%)	22	30	0.42
Female	42 (44.7%)	16	26	
Refugee status, *n* (%)				
Yes	54 (57.4%)	18	36	0.079
No	40 (42.6%)	20	20	
Body Mass Index, mean ± SD	28.48 ± 5.3	29.14 ± 5.09	28.03 ± 5.43	0.321
Education level, *n* (%)				
Illiterate	12	5	7	0.060
Less than high school	48 (63.8%)	16	32	
High school	20 (21.3%)	6	14	
More than high school degrees	14 (14.9%)	11	3	
Marital status, *n* (%)				
Married	68 (72.3%)	28	40	0.707
Widowed	17 (18.1%)	6	11	
Single	5 (5.3%)	3	2	
Divorced	4 (4.3%)	1	2	
Smoking, *n* (%)				
Yes	28 (29.8%)	10	18	0.355
No	66 (70.2%)	28	38	
Hypertension *n* (%)				
Yes	64 (68.1%)	26	38	0.569
No	30 (31.9%)	12	18	
Diabetes Mellitus *n* (%)				0.520
Yes	51 (54.3%)	21	31	
No	51 (54.3%)	17	26	
Heart disease *n* (%)				0.237
Yes	35 (37.2%)	12	23	
No	59 (72.8%)	26	33	
Cholesterol *n* (%)				0.494
Yes	21 (22.3%)	9	12	
No	71 (87.7%)	29	44	
First or recurrent stroke				
First stroke *n* (%)	72 (76.6%)	29	43	0.574
Recurrent stroke *n* (%)	22 (23.4%)	9	13	
Duration since the last stroke (in months), mean ± SD	36.8 ± 47.56	39.2 ± 59.2	35.05 ± 37.94	0.769
Chronicity of stroke *n* (%)				
Acute stroke	6 (6.5%)	1	5	0.138
Sub-acute stroke	7 (7.5%)	4	3	
Chronic stroke	80 (86%)	33	47	
Hemiplegic side, *n* (%)				
Left	37 (39.4%)	12	25	0.145
Right	57 (60.6%)	26	31	

**Table 2:** The correlation between the Pittsburg Sleep Quality Index (PSQI) and the Shoulder Pain and Disability Index (SPADI).

SPADI/PSQI	PSQI Subjective QOS	PSQI Sleep Latency	PSQI Sleep Duration	PSQI Sleep Efficiency	PSQI Sleep Disturbance	PSQI Medications	PSQI Daytime Dysfunction	PSQI Global Score
SPADI pain	0.35 **	−0.01	−0.04	−0.041	0.38 **	0.08	0.37 **	0.27 **
SPADI disability	0.14	−0.144	−0.21 **	−0.111	0.16	−0.11	0.25 *	−0.06
SPADI total	0.28 **	−0.118	−0.19	−0.11	0.36 **	−0.05	0.30 **	0.06

* Correlation is significant at the 0.01 level; ** correlation is significant at the 0.05 level. QOS, Quality of sleep.

**Table 3:** The correlation between the Pittsburgh Sleep Quality Index components and the total score and stroke outcomes (impact of stroke, fatigue, and quality of life).

	PSQI Subjective QOS	PSQI Sleep Latency	PSQI Sleep Duration	PSQI Sleep Efficiency	PSQI Sleep Disturbance	PSQI Medications	PSQI Daytime Dysfunction	PSQI Global Score
i-Stroke Impact Scale (SIS)								
ADL	−0.216 *	0.224 *	0.241 *	0.225 *	−0.247 *	−0.261 *	−0.475 **	−0.084
Memory	−0.011	−0.019	0.269 **	0.046	−0.211 *	−0.449 **	−0.326 **	−0.139
Emotion	−0.065	0.010	0.013	−0.141	−0.152	−0.189	−0.241 *	−0.181
Communication	−0.081	0.134	0.234 *	0.159	−0.234 *	−0.458 **	−0.368 **	−0.111
Social participation	−0.253 *	0.042	0.170	0.070	−0.217 *	−0.042	−0.471 **	−0.159
Stroke recovery	r = −0.317 **	−0.062	0.200	0.095	−0.151	−0.385 **	−0.472 **	−0.268 **
ii-Modified Fatigue Impact Scale (MFIS)								
Physical fatigue	0.282 **	0.071	−0.040	0.038	0.200	0.132	0.429 **	0.286 **
Cognitive fatigue	0.291 **	0.196	−0.063	0.139	0.247 *	0.339 **	0.468 **	0.416 **
Psychosocial fatigue	0.329 **	0.076	−0.414	0.026	0.232 *	0.132	0.411 **	0.283 **
MFIS total score	0.329 **	0.152	−0.064	0.099	0.256 *	0.268 **	0.505 **	0.397 **
iii-Medical Outcomes Short Form 12 (SF-12)								
Physical SF-12	−0.151	0.007	0.133	0.145	−0.177	−0.114	−0.489 **	−0.156
Mental SF-12	−0.210 *	−0.176	0.085	−0.037	−0.185	−0.104	−0.366 **	−0.260 *
Total SF-12	−0.222 *	−0.126	0.123	0.039	−0.216 *	−0.127	−0.490 **	−0.261 *

* Correlation is significant at the 0.01 level; ** correlation is significant at the 0.05 level. QOS, Quality of sleep.

## Data Availability

Data available on request from the corresponding author.
